# The successful introduction of an adapted form of the mini extra corporeal circulation used for cardiac surgery in an obese patient

**DOI:** 10.1186/1749-8090-7-20

**Published:** 2012-03-09

**Authors:** Patrizio Sartini, Anna Winfield, Federico Bizzarri

**Affiliations:** 1 Department of Medical Surgical Sciences and Biotechnologies, Cardiac Surgery Unit, Polo Pontino, Universita' degli Studi di Roma "Sapienza", Latina, Italy; 2 Department of Cardiac Surgery, Yorkshire Heart Centre, Leeds General Infirmary, Great George Street, LS1 7EH Leeds, UK

**Keywords:** Cardiopulmonary bypass, Obese patients, Aortic valve replacement, Mini extra corporeal circulation

## Abstract

Obese patients undergoing cardiac surgery have been shown to have a high risk of developing postoperative complications, specifically: increased length of hospital stay, readmission to intensive care unit, acute renal failure, deep sternal wound infections and new onset of atrial fibrillation.

A custom-made circuit was created to allow the use of Mini Extra Corporeal Circulation (MECC) but permitting the switch to a closed siphon drainage system in the case of difficulties.

To limit artificial surface contact a small oxygenating device (Admiral, Eurosets) was employed in spite of the patients size. This adapted circuit permits a feasible and safer approach to using MECC. This report suggests that smaller oxygenators could be integrated into clinical practice in an adult MECC configuration, even for more obese patients, limiting artificial surface contact.

## Background

Obese patients undergoing cardiac surgery have an increased risk of developing specific postoperative complications. Obesity (defined as BMI > 30) is associated with prolonged mechanical ventilation, readmission to intensive care unit, increased rates of deep sternal wound infection and a length of stay greater than 14 days. Both obesity and morbid obesity, especially when other risk factors such as diabetes mellitus and hypertension are present, may increase the rate of onset of acute renal failure. Nevertheless, an increased BMI does not increase the risk of early postoperative death or the risk of postoperative cerebrovascular accidents and the rates of postoperative bleeding in obese patients are significantly lower [1]. The profile of patients undergoing cardiac surgery has changed in recent times, as rates of obesity increase, the proportion of obese patients requiring cardiac surgery has increased also.

The aim of this article is to propose a progressive approach to MECC, demonstrating its successful use in an obese patient.

## Case presentation

A 73 year-old man was admitted to our unit with severe aortic stenosis requiring aortic valve replacement. The patient had numerous risk factors for cardiovascular disease including obesity (BMI = 40; BSA = 2,46), hypercholesterolemia, hypertriglyceridemia, Type II Diabetes Mellitus and being a cigarette smoker. The procedure was performed through a J-shaped mini-sternotomy [2] with routine ascending aorta and right atrial cannulation. After opening the aorta, we carefully removed all the debris on the valvular annulus that extended to the mitro-aortic junction. A tissue valve was then implanted. The operation was completed routinely and without incident. The patient was discharged from the Intensive Care Unit on post-operative day two. There were no major complications.

## Method

Cardio Pulmonary Bypass (CPB) was performed using a closed custom-made circuit designed to perform Mini-extracorporeal circulation (MECC). The circuit was designed such that the system could switch to a conventional closed siphon drainage system if necessary.

**Figure 1:**
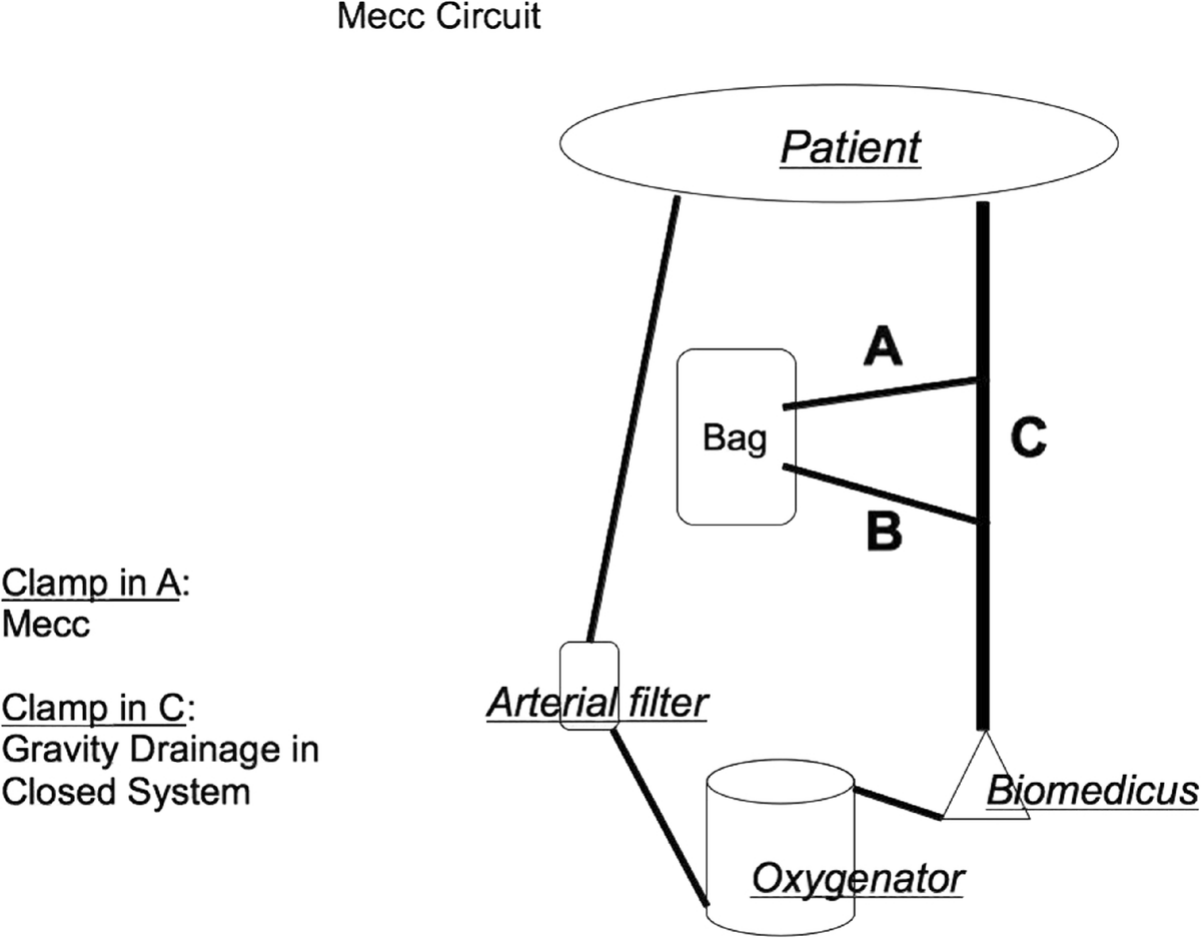
**Mecc Circuit**.

A schematic description of our circuit can be seen in Figure 1. The circuit consisted of a 1/2 inch venous line (compatible for siphon drainage) and 3/8 inch arterial line, a collapsible bag was interposed in the venous line and was connected to it by two 3/8 inch short pipes, the 1/2 inch venous line then met a centrifugal pump (Biopump Medtronic). After the venous section there was the oxygenator (Eurosets, Mirandola, Italy), the arterial filter and the final 3/8 inch segment of the arterial pipe.

This dual purpose circuit employed a 1/2 inch venous pipe longer than the ones usually used in MECC and the same pipe was selected for use as the venous cannula suitable for siphon drainage (24 fr Aortic cannula; 44/36 fr right atrium cannula. DLP^® ^Medtronic, Inc.). To minimise artificial surface contact and improve biocompatibility we employed the Admiral^® ^oxygenator (Eurosets, Mirandola, Italy), which had the smallest available membrane and phosphorylcoline treatment of the lines and oxygenator. It is an integrated microporous hollow-fibre oxygenator, with a total membrane surface area of 1.35 m^2^, a heat exchanger surface area of 0,08 m^2 ^and a low prime volume (190 ml) [3]. The oxygenator and the circuit had a superficial phosphorylcoline (Pc) coating. This coating is reported to provide some protection against blood clot activation [4]. The circuit was placed under the operating table to obtain a drainage pressure of approximately -25, mmHg. A pressure transducer monitored the negative pressure in venous line. Retrograde Autologous Priming (RAP) was employed to limit haemodilution [5].

Buckberg cardioplegic solution was delivered directly into the coronary ostia and into the coronary sinus during aortotomy. Mild hypothermia was induced. An auto-transfusion device (Electa, Soring Group, Italy) was used to collect, wash and store shed red blood cells, the remaining circuit perfusate was also treated by Electra and autologous red cell transfusion was initiated in the post-operative period [5]. Hyperglycaemia was managed according to the modified Portland protocol.

Oxygenator performance was evaluated by monitoring of serial arterial blood gas measurments (pO_2 _150-200 mmHg). Adequacy of perfusion was monitored by Oxygen Delivery (DO_2_), lactate production and the ratio between the oxygen delivery and carbon dioxide production (DO_2_/VCO_2_). The DO_2 _formula can be calculated as follows: [pump flow × (Hb × O_2_Sat × 1,36) + 0,003 × PaO_2 _] [6]. Blood lactate monitoring is more appropriate for detecting the correct matching of oxygen supply and demand during CPB [7].

Carbon dioxide production (VCO_2_) was calculated as follows: VCO_2 _indexed (mL × min -1 × m-2): eCO_2 _(mmHg) × Ve (L/min) × 1000/760 × BSA (m^2^). Exhaled carbon dioxide from oxygenator (CO_2_) and gas flow into the oxygenator (Ve) were simultaneously recorded at each sampling aspiration.

## Conduct of perfusion

Tubing clamps were placed in both A and C 3/8 inch pipes, starting from the venous bag. The centrifugal pump velocity was previously set to overcome the patient's systemic blood pressure avoiding back flow. At the onset of CPB the clamp in position A was partially opened obtaining drainage from the patient into a venous bag. Once the partial release of the "A segment clamp" permitted the venous drainage, the blood collected in the collapsible bag began to flow from the centrifugal pump and gradually increased with further release of the "A clamp segment". With this clamp configuration siphon drainage was obtained as it occurs in conventional closed system. In few minutes full CPB was established. At this point, in order to switch toward MECC, the "C segment clamp" was released and subsequently the "A segment" was clamped.

At this moment there was a transient diminution of systemic perfusion due to volume collection in the collapsible bag but this was readily corrected with light occlusion of the "C segment" until the collapsible bag emptied returning perfusate, restoring circulating volume.

Once the circulating volume correction was performed the desired flow was obtained using MECC conduction. In this kinetic assisted drainage configuration with a closed circuit the monitoring of central venous pressure (CVP) was helpful in the maintenance of desired blood flow and avoided over-distension of the right atrium. Excessive negative pressure and venous line chattering was overcome by partial clamping of the 1/2 inch venous line. The negative pressure in 1/2 inch venous line was kept to approximately -50 mmHg.

## Discussion

MECC optimizes the extracorporeal circulation, thus resulting in a reduction in the inflammatory reaction, blood exposure and trauma [8]. The use of MECC as demonstrated in this study could be incorporated into daily practice without considerable difficulty at other centres [9] as this centre has done following the experience of others.

The use of the venous bag included in the circuit permits a rapid and safe switch from MECC to a closed conventional extracorporeal circulation. The oxygenator (Eurosets, Mirandola, Italy) performed satisfactorily throughout the procedure despite the small oxygenating surface [10]. To our knowledge this case represents the patient with the largest body mass index to have been treated with this small oxygenating device.

**Figure 2:**
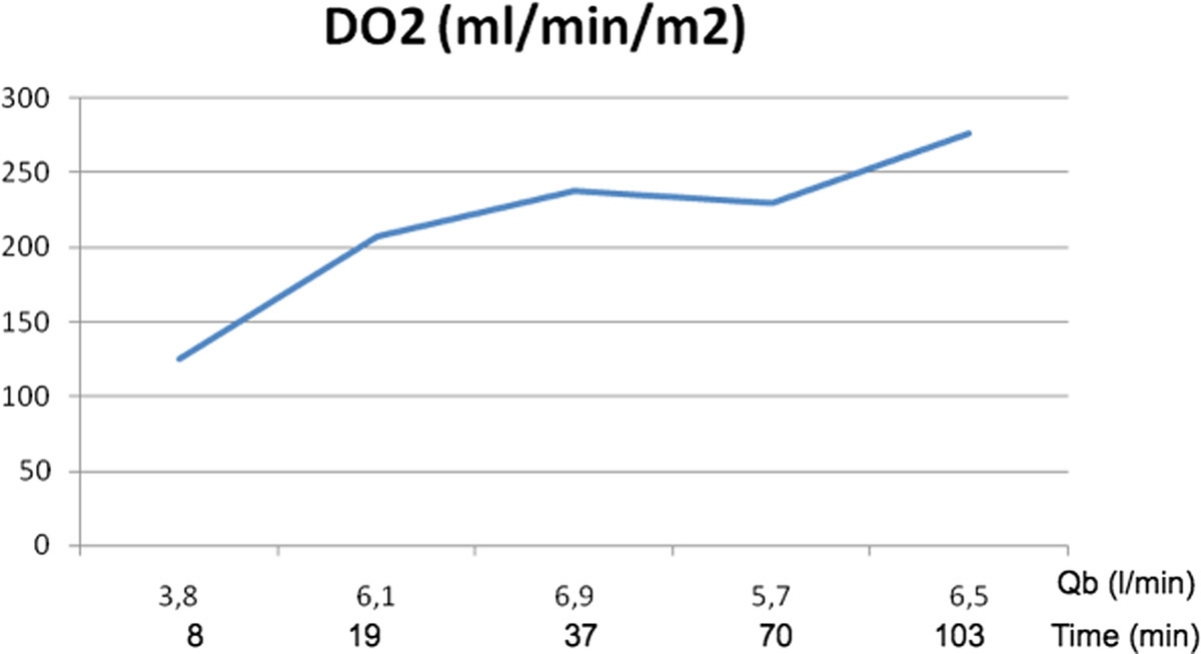
**Intra-Operative Oxygen Delivery: (DO_2_)**.

**Figure 3:**
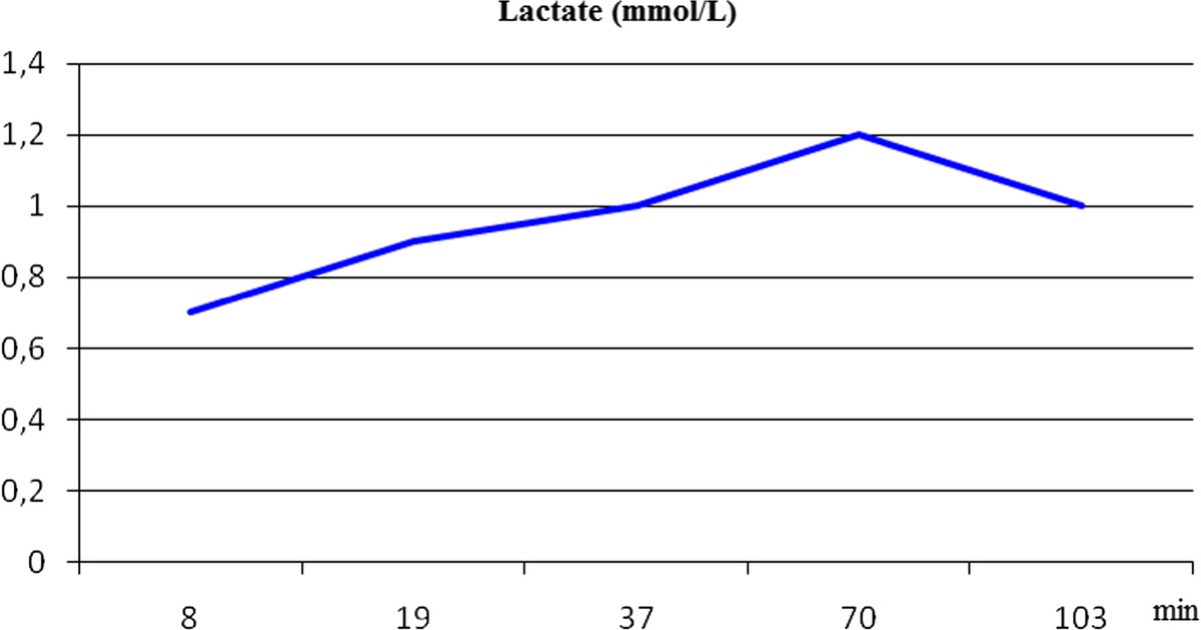
**Intra-Operative Lactate Production**.

**Figure 4:**
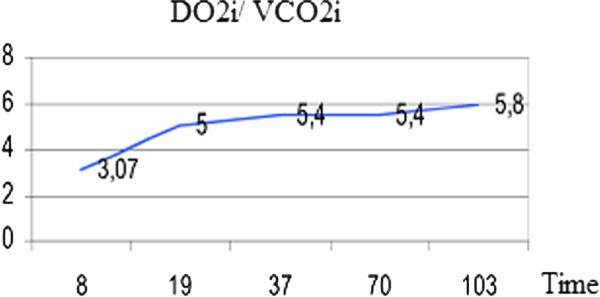
**Oxygen Delivery/Carbon Dioxide Production: (DO_2_/VCO_2_)**.

Adequacy of perfusion was monitored by Oxygen Delivery (DO_2_) [Figure 2], lactate production [Figure 3] and the ratio between the oxygen delivery and carbon dioxide production (DO_2_/VCO_2_) [11,12] [Figure 4]. A DO_2_/VCO_2 _ratio of < 5 is thought to be predictor of hyperlactatemia and although not specific, an increase or change in lactate level during Cardiopulmonary bypass, may be a marker of regional hypoperfusion or increased metabolic demand [13]. In our case we ran a lower DO_2 _than suggested in previous studies. Ranucci et al. fixed a critical value of 270 ml/min/m^2 ^suggesting that a lower value could induce a condition of circulatory shock [14,15]. In our patient a low DO_2 _value, well matched carbon dioxide parameters showing a value higher than 5 as result of the thermic strategy. Since the procedure was not normothermic we choose to pump at a lower Ht and trigger the transfusion point according to the oxyphoretic needs.

The intra-operative [Figure 3] lactate production remained within a normal range throughout the procedure, providing reassurance about the adopted perfusion strategy, which permitted a prudent transfusion policy.

**Figure 5:**
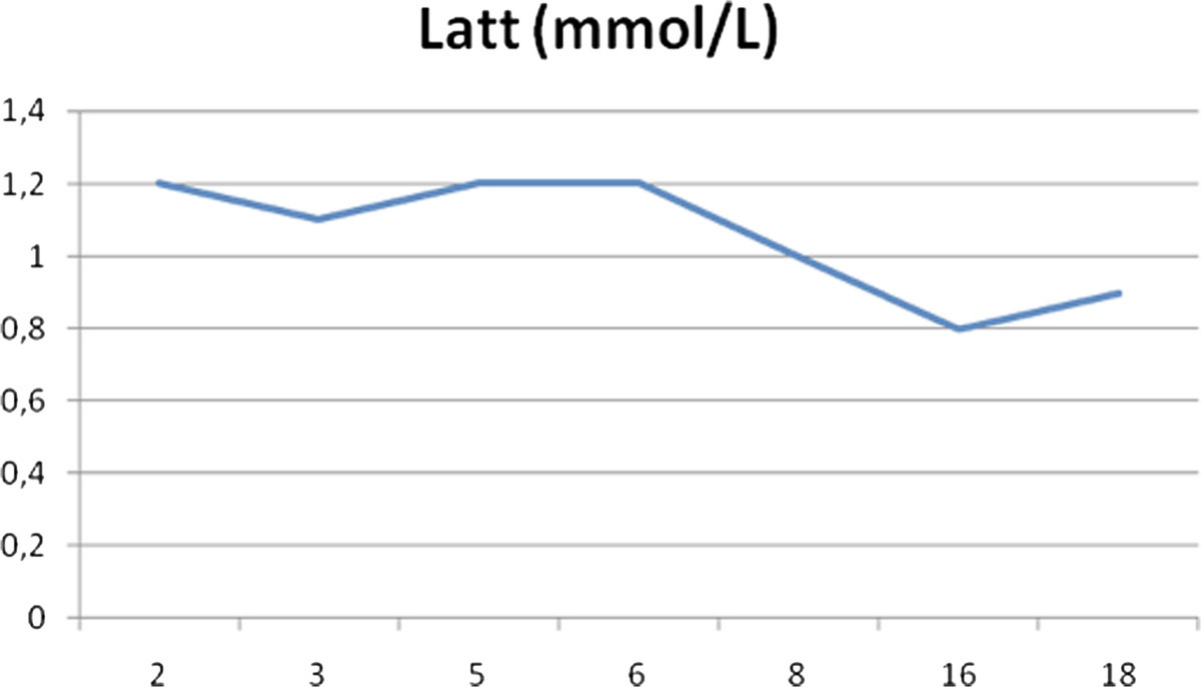
**Post Operative Lactate Production**.

The lactate production monitored up to 18 hours post operatively [Figure 5] followed a normal course.

## Conclusion

With this simple circuit the surgical team can gain the necessary confidence in MECC and experience can be gained safely. The integration of a smaller oxygenator than usual in a MECC circuit, could be a positive contribution in the continuous effort to increase perfusion optimization [16-19] and manufacturing companies could consider this for their own commercial development. The use of MECC in this way has been shown in this case to be successful in the obese patient.

## Competing Interests

The authors declare that they have no competing interests.

## Consent

Written informed consent was obtained from the patient for publication of this report and any accompanying images.

## Disclosure

The authors do not receive honoraria, consultation fees or support from cited companies.

## Authors' Contributions

PS and FB conceived the new circuit and AW participated in the design of the study. All authors read and approved the final manuscript.
